# Multiple Coronary-Cameral Fistulae Draining Exclusively Into the Left Atrium Associated With Atrial Thrombosis

**DOI:** 10.7759/cureus.110752

**Published:** 2026-06-12

**Authors:** Daniela Cordoba-Alvarado, Citlalli Diaz-Leal, Cristian J Sosa-Alvarez, Juan C Macias-Hernandez, Carlos Jimenez-Lopez

**Affiliations:** 1 Cardiology, Instituto de Seguridad y Servicios Sociales de los Trabajadores del Estado (ISSSTE), Zapopan, MEX; 2 Cardiology, Hospital Regional Dr. Valentin Gomez Farias Instituto de Seguridad y Servicios Sociales de los Trabajadores del Estado (ISSSTE), Zapopan, MEX

**Keywords:** atrial thrombosis, coronary artery fistula (caf), coronary-cameral fistula, multimodality cardiac imaging, spontaneous echocardiographic contrast

## Abstract

Coronary artery fistulae (CAF) represent uncommon anomalous connections linking the coronary arterial tree directly to cardiac chambers or great thoracic vessels. Termination into the left atrium is extraordinarily rare, especially when presenting as multiple fistulous tracts. We describe the case of a 60-year-old woman with permanent atrial fibrillation, previous mechanical mitral valve replacement, recurrent systemic thromboembolism, and prosthetic valve thrombosis. Coronary angiography incidentally revealed two coronary-cameral fistulae arising from the proximal left circumflex artery and a third fistula originating from the right coronary artery's distal conus branch, with all channels draining exclusively into the left atrial cavity.

Transesophageal echocardiography confirmed continuous turbulent flow entering the left atrium, accompanied by dense spontaneous echocardiographic contrast (SEC) and an organized mural left atrial thrombus. The combination of permanent atrial fibrillation, chronic left atrial volume overload driven by the fistulous shunt, prior mitral valve pathology, and inadequate anticoagulation likely generated a highly prothrombotic environment.

This case emphasizes the critical role of multimodality cardiac imaging in characterizing intricate coronary-cameral architecture. Furthermore, it demonstrates that left atrial drainage fistulae may have contributed to atrial flow disturbance and thrombotic propensity in the context of multiple established thromboembolic risk factors, even without achieving conventional thresholds for a hemodynamically significant shunt.

## Introduction

Coronary artery fistulae (CAF) are anomalous communications that directly connect the coronary arteries to heart chambers or major thoracic vessels. Specifically, those that drain into a cardiac cavity are termed coronary-cameral fistulae [1]. These anomalies can originate from any of the three main coronary vessels, including the left main coronary artery [2].

The incidence of CAF in the general population is approximately 0.002%. About 20% of affected individuals present with concomitant congenital heart diseases, most frequently associated with pulmonary or aortic atresia and patent ductus arteriosus [3].

While coronary fistulae are generally solitary, multiple fistulae represent a rare anatomical variant [4]. The right ventricle is the most common drainage site (approximately 40%), followed by the right atrium and pulmonary artery [5]. Drainage into the coronary sinus is reported in approximately 7% of cases, while drainage into the left atrium, as seen in the present case, occurs in only 5% of instances [6]. ​The right coronary artery is the most frequent vessel of origin, followed by the left anterior descending artery, whereas the left circumflex artery is less commonly involved [7]. The rarity of the present case lies in the simultaneous occurrence of three uncommon clinical features: the origin of the fistulae in the left circumflex artery and the right coronary artery, the presence of multiple communications, and an exceptional bilateral drainage site into the left atrium. Furthermore, the coexistence of this anomaly with mitral valve disease and subsequent prosthetic dysfunction adds a layer of hemodynamic complexity, as the coronary-cameral shunt directly impacts the left atrial volume and pressure dynamics. While solitary coronary-cameral fistulae draining into the left atrium have been occasionally described in larger cohorts, the simultaneous occurrence of multiple fistulous tracts with bilateral origin in this anatomical setting remains exceptional [1,6]. To our knowledge, this specific combination of findings is sparsely reported in the literature, highlighting the importance of multimodality imaging and invasive angiography in its diagnosis and management.

## Case presentation

**Figure 1:**
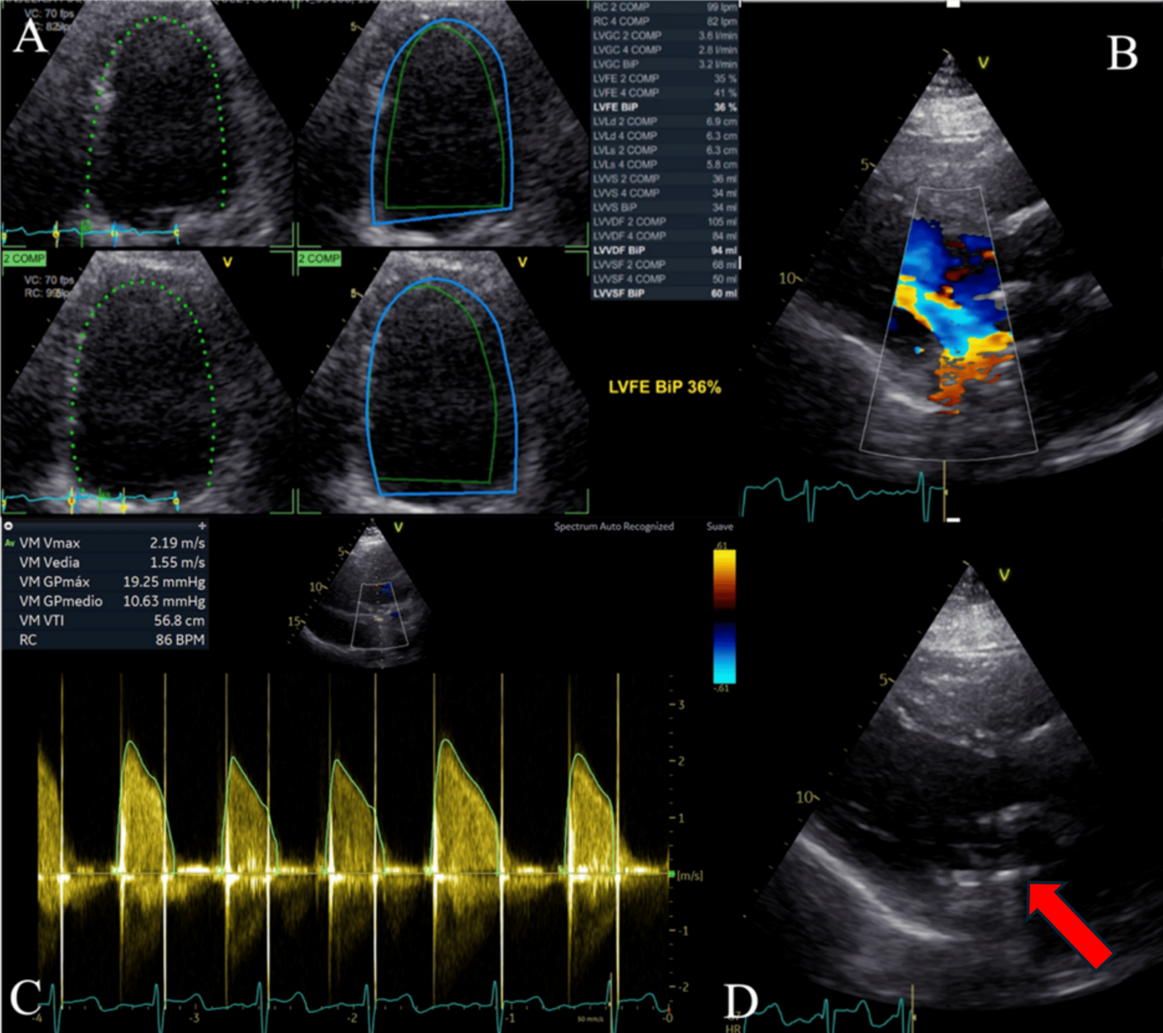
Transthoracic echocardiographic assessment of prosthetic mitral valve thrombosis. Panel A demonstrates quantitative left ventricular systolic function assessment by the Simpson biplane method, showing reduced left ventricular ejection fraction (LVEF, 36%). Panel B shows color Doppler interrogation across the mitral bioprosthesis, demonstrating turbulent transmitral flow. Panel C demonstrates continuous-wave Doppler across the prosthetic mitral valve, revealing elevated transprosthetic gradients (mean gradient, 10.63 mmHg; peak gradient, 19.25 mmHg) and increased transvalvular velocity (Vmax: 2.19 m/second), findings suggestive of prosthetic valve dysfunction. Panel D shows transthoracic echocardiography demonstrating restricted mobility of the anterior prosthetic hemi-disk (red arrow), consistent with prosthetic valve thrombosis.

A 60-year-old woman presented with a complex medical history characterized by recurrent multi-territorial thrombotic events. Her background included a history of childhood rheumatic fever, hypertension, type 2 diabetes, permanent atrial fibrillation, and a prior ischemic stroke. She subsequently underwent percutaneous balloon mitral valvuloplasty. Notably, due to poor adherence to oral anticoagulation therapy, she developed left renal and splanchnic arterial thrombosis. She later underwent mechanical mitral valve replacement (St. Jude number 29). Two years after the initial surgery, she developed acute heart failure symptoms. A transthoracic echocardiogram revealed restricted mobility of the anterior hemi-disk and elevated gradients, consistent with prosthetic valve thrombosis (Figure 1).

**Figure 2:**
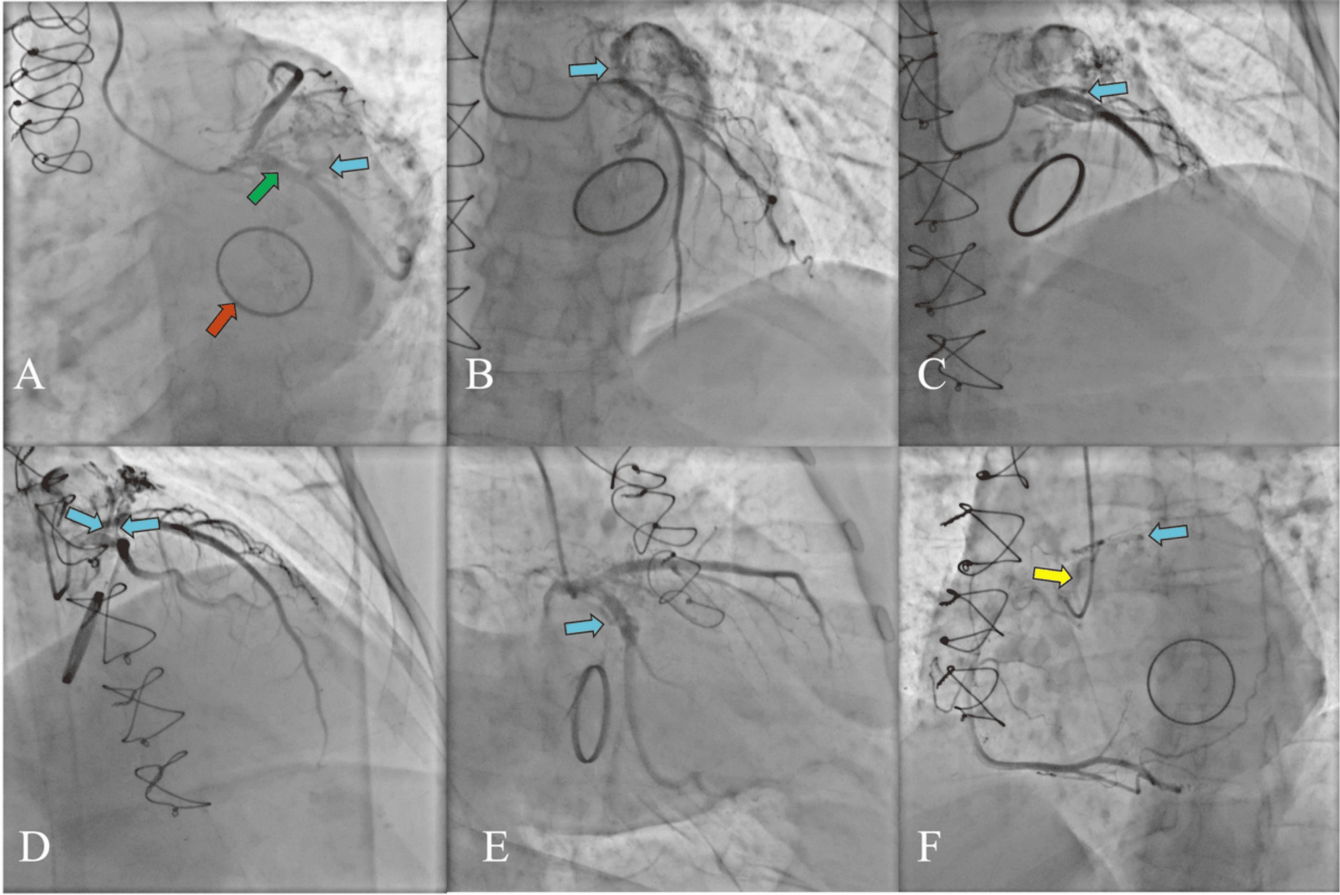
Coronary angiography demonstrating multiple coronary-cameral fistulae draining into the left atrium. Panels A-E correspond to selective angiography of the left coronary system, while panel F shows selective right coronary angiography. In panel A, the green arrow identifies the left circumflex artery (LCx), from which multiple tortuous fistulous communications arise (blue arrows) with drainage toward the left atrium. The orange arrow indicates the mechanical mitral prosthesis (St. Jude valve). Panels B-E further delineate the anomalous fistulous network originating from the proximal LCx (blue arrows), demonstrating opacification and drainage into the left atrial chamber. Panel F corresponds to right coronary angiography; the yellow arrow identifies the conus branch, from which an additional coronary-cameral fistula originates (blue arrow) with drainage into the left atrium.

During the diagnostic workup, coronary angiography identified two small-caliber coronary-cameral fistulae originating from the proximal segment of the left circumflex artery and an additional fistula originating from the right coronary artery, specifically localized at the distal segment of the conus branch, all of them draining into the left atrium (Figure 2). Visually, all fistulous tracts were of small size, with an estimated diameter smaller than the diagnostic angiography catheter (less than 2 mm).

The remaining coronary arteries showed no significant angiographic lesions. Subsequently, the patient underwent a second mitral valve replacement with a bioprosthetic valve (Abbott Epic Plus 27 mm) and left atrial appendage closure.

**Figure 3:**
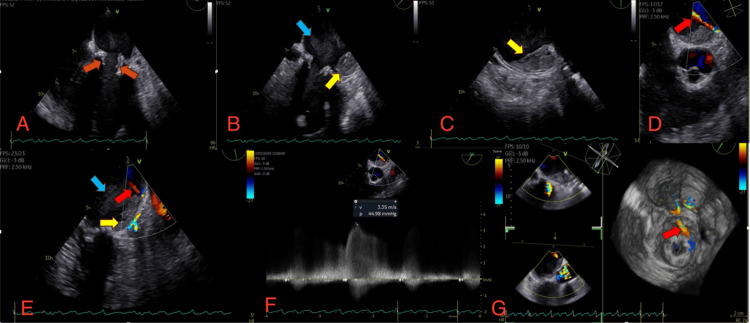
Transesophageal echocardiographic findings demonstrating left atrial thrombosis and coronary-cameral fistulous drainage into the left atrium. Panel A shows the Abbott bioprosthetic mitral valve with adequate leaflet opening (orange arrows). Panel B demonstrates dense spontaneous echocardiographic contrast within the left atrium (blue arrow) and an organized mural thrombus attached to the atrial wall (yellow arrow). Panel C further delineates the left atrial thrombus (yellow arrow). Panel D shows the coronary-cameral fistula (red arrow) adjacent to the aortic valve (center), with color Doppler demonstrating the fistulous tract draining into the left atrium. Panel E depicts the left atrium and left ventricle; the blue arrow indicates spontaneous echocardiographic contrast, the red arrow identifies the fistulous flow, and the yellow arrow indicates the atrial thrombus. Panel F demonstrates the continuous-wave Doppler profile of the fistulous flow velocity. Panel G shows three-dimensional transesophageal echocardiographic reconstruction, with the red arrow highlighting the fistulous tract draining into the left atrium.

At a subsequent visit, the patient reported a one-month history of lipothymia and New York Heart Association (NYHA) class III dyspnea. Bioprosthetic valve dysfunction was suspected, prompting a follow-up transesophageal echocardiogram (TEE), which revealed a dilated left atrium with dense spontaneous echocardiographic contrast (SEC) and evidence of an organized parietal thrombus adhered to the anterolateral and anterior walls of the atrium. No mobile elements or interference with the bioprosthetic valve function was identified. However, during the transesophageal study, in addition to the atrial thrombus, the presence of a continuous flow entering directly into the left atrium originating from the left circumflex artery was identified, which was classified as a coronary-cameral fistula (Figure 3 and Video 1).

**Video 1:** Transesophageal echocardiogram demonstrating coronary-cameral fistulous flow, dense spontaneous echo contrast, and left atrial thrombosis. The video clip demonstrates the continuous and turbulent flow originating from the coronary-cameral fistula and entering directly into the left atrial cavity adjacent to the aortic valve. Additionally, it highlights the severe blood stasis that manifested as dense, smokelike spontaneous echocardiographic contrast (SEC) within the dilated left atrium, alongside the large, organized, and immobile mural thrombus adhered to the atrial wall.

The systemic blood flow (Qs)/pulmonary blood flow (Qp) ratio calculated via echocardiography was 1.2; since the circumflex fistulae drain into the left atrium, the Qp/Qs ratio did not exceed 1.5.

The patient was discharged in stable condition with outpatient cardiology follow-up arranged. Bioprosthetic valve dysfunction was ultimately excluded based on the echocardiographic findings. Anticoagulation therapy was optimized by transitioning the patient to a direct oral anticoagulant (DOAC) regimen with rivaroxaban (20 mg once daily), which was favored over vitamin K antagonists (VKA) to mitigate the high risk of recurrent nonadherence and suboptimal monitoring previously documented. This decision was tailored to the persistent thrombotic risk imposed by the combination of permanent atrial fibrillation, prior systemic thromboembolism, and chronic left atrial volume loading from the fistulous drainage. The patient was counseled regarding warning signs requiring urgent reassessment, and the case was discussed in a multidisciplinary setting to evaluate the potential benefit of elective percutaneous fistula closure, weighing the hemodynamic contribution of the shunt against the elevated surgical risk in the context of two prior open cardiac surgeries.

## Discussion

As previously mentioned, the incidence of CAF is extremely low at 0.002% [3]. Our case is particularly distinctive because drainage into the left atrium has been reported in only a small minority of cases, underscoring the exceptional anatomical configuration observed in this patient [6].

During embryological development, the trabeculated myocardium contains sinusoidal spaces communicating with the coronary arteries, veins, and cardiac chambers. With progressive myocardial maturation, these channels normally regress and form the Thebesian venous system. The failure of this involution process results in the persistence of the sinusoidal communications and the subsequent development of coronary artery fistulae [8].

Coronary artery fistulae can be characterized noninvasively using echocardiography. Transthoracic echocardiography with color Doppler is useful for identifying proximal fistulous flow, although visualization may be limited in distal coronary segments. Transesophageal echocardiography provides superior spatial resolution and closer proximity to the structures of interest, allowing more accurate delineation of the fistula origin, course, and drainage chamber [9]. Multiplane imaging further facilitates comprehensive anatomical assessment. Continuous turbulent Doppler flow at the drainage site represents the most characteristic echocardiographic finding, although intermittent diastolic flow may also be encountered [10]. In our patient, transesophageal echocardiography demonstrated continuous turbulent flow adjacent to the left coronary cusp with direct drainage into the left atrium.

Spontaneous echocardiographic contrast (SEC) reflects blood stasis and is widely recognized as a precursor to thrombus formation. SEC is frequently associated with mitral stenosis, low cardiac output states, left atrial enlargement, and reduced mitral valve area [11]. In the present case, these predisposing factors were compounded by permanent atrial fibrillation and the chronic hemodynamic burden imposed by the coronary-cameral fistulae. Continuous left atrial volume loading likely promoted progressive chamber dilation and flow deceleration, generating a markedly prothrombotic atrial environment. In this context, the fistulous drainage likely acted synergistically with other well-established factors in this patient, such as permanent atrial fibrillation, rheumatic atrial myopathy, a history of prosthetic valve thrombosis, and previous inadequate anticoagulation, to amplify the thrombotic propensity. In this setting, dense SEC should not be interpreted as an incidental echocardiographic finding but rather as a dynamic manifestation of ongoing thrombogenic activity requiring strict anticoagulation optimization.

Clinical manifestations of CAF vary according to fistula size and hemodynamic impact, ranging from asymptomatic presentations in nearly 75% of patients to myocardial ischemia, arrhythmias, valvular dysfunction, and congestive heart failure [1]. Arrhythmias may develop secondary to chronic chamber dilation [1]. Although our patient had permanent atrial fibrillation, its pathogenesis was likely multifactorial, related not only to the coronary-cameral fistulae but also to rheumatic mitral valve disease, advanced age, left atrial enlargement, and prior cardiac surgery. Consequently, this multifactorial arrhythmia directly interacted with her history of prosthetic valve thrombosis, rheumatic atrial myopathy, and periods of inadequate anticoagulation, serving as synergistic mechanisms that substantially amplified her overall thrombotic propensity.

Current treatment recommendations favor intervention in symptomatic patients, in the presence of hemodynamically significant shunts (Qp/Qs >1.5), myocardial ischemia secondary to the coronary steal phenomenon, progressive chamber enlargement, or the risk of complications such as thrombosis or rupture [12]. Available therapeutic strategies include surgical ligation and transcatheter embolization, with treatment selection guided by anatomical characteristics, institutional expertise, and individual surgical risk [12]. Conventional shunt assessment is based on the Qp/Qs ratio, where values greater than 1.5 define a significant left-to-right shunt [12]. However, this paradigm assumes increased pulmonary blood flow relative to systemic flow, an assumption that does not apply when fistulous drainage occurs exclusively into the left atrium [1,5].

In our case, the fistulae created a left-to-left shunt in which blood originating from the coronary circulation returned directly into the left atrium without traversing the pulmonary vascular bed. Consequently, pulmonary blood flow (Qp) remained largely unchanged because the fistulous drainage bypassed the pulmonary circulation and returned directly into the left atrium, limiting the physiological applicability of the conventional Qp/Qs framework in this anatomical setting. For this reason, shunt magnitude was expressed as a Qs/Qp ratio, which measured 1.2 in our patient, indicating a modest increase in systemic recirculation without pulmonary overcirculation. Although this value would not traditionally meet the criteria for intervention, the clinical implications of chronic left atrial volume loading extend beyond conventional hemodynamic thresholds [12]. Persistent atrial overload may amplify chamber dilation, worsen flow stasis, and potentiate thrombus formation, particularly in patients with concomitant atrial fibrillation, prior valve surgery, and suboptimal anticoagulation adherence [1,11].

Within this broader pathophysiological framework, the coexistence of recurrent systemic thromboembolism, dense SEC, and organized left atrial thrombus raises the possibility that fistula closure could reduce an additional substrate for atrial stasis. If intervention was ultimately considered, percutaneous transcatheter embolization would likely represent the preferred strategy given the patient's prior surgical history and elevated operative risk after two previous open cardiac procedures.

## Conclusions

This case illustrates the diagnostic and therapeutic complexity of multiple coronary-cameral fistulae originating from the left circumflex and right coronary arteries with exclusive drainage into the left atrium. Beyond simple anatomical identification, multimodality imaging, specifically the integration of coronary angiography and transesophageal echocardiography, proved essential in elucidating the hemodynamic impact of these shunts and their role as pivotal modulators of atrial stasis and thrombus formation.

Crucially, in patients harboring coronary-cameral fistulae draining into the left atrium, the chronic left atrial volume overload imposed by the shunt should be recognized as an independent thrombotic risk factor, one capable of lowering the threshold for intervention even in the absence of hemodynamically significant shunt ratios by conventional criteria. Structural anomalies and biological thrombotic risk factors are not mutually exclusive and must be investigated in parallel. Ultimately, a comprehensive and individualized approach, one that simultaneously addresses the anatomical shunt, the systemic hypercoagulable state, and the downstream consequences of chronic volume loading, is paramount to optimizing outcomes in this rare and underrecognized clinical scenario.
